# Evaluation of a high resolution diode array for CyberKnife quality assurance

**DOI:** 10.1002/acm2.14053

**Published:** 2023-05-29

**Authors:** Juan D. García‐Fuentes, David Sevillano, Rafael Colmenares, Ana B. Capuz, Rafael Morís, Miguel Cámara, Pablo Galiano, Sandra Williamson, María J. Béjar, Daniel Prieto, Feliciano García‐Vicente

**Affiliations:** ^1^ Department of Medical Physics Hospital Universitario Ramón y Cajal, IRyCIS Madrid Spain; ^2^ Department of Radiology Rehabilitation and Physiotherapy Universidad Complutense de Madrid Madrid Spain

**Keywords:** CyberKnife, diode array, quality assurance, radiochromic film

## Abstract

**Purpose:**

The CyberKnife quality assurance (QA) program relies mainly on the use of radiochromic film (RCF). We aimed at evaluating high‐resolution arrays of detectors as an alternative to films for CyberKnife machine QA.

**Methods:**

This study will test the SRS Mapcheck (Sun Nuclear, Melbourne, Florida, USA) diode array and its own software, which allows three tests of the CyberKnife QA program to be performed. The first one is a geometrical accuracy test based on the delivery of two orthogonal beams (Automated Quality Assurance, AQA). Besides comparing the constancy and repeatability of both methods, known errors will be introduced to check their sensitivity. The second checks the constancy of the iris collimator field sizes (Iris QA). Changes in the field sizes will be introduced to study the array sensitivity. The last test checks the correct positioning of the multileaf collimator (MLC). It will be tested introducing known systematic displacements to whole banks and to single leaves.

**Results:**

The results of the RCF and diode array were equivalent (maximum differences of 0.18 ± 0.14 mm) for the AQA test, showing the array a higher reproducibility. When known errors were introduced, both methods behaved linearly with similar slopes. Regarding Iris QA, the array measurements are highly linear when changes in the field sizes are introduced. Linear regressions show slopes of 0.96–1.17 with *r* ^2^ above 0.99 in all field sizes. Diode array seems to detect changes of 0.1 mm. In MLC QA, systematic errors of the whole bank of leaves were not detected by the array, while single leaf errors were detected.

**Conclusions:**

The diode array is sensitive and accurate in the AQA and Iris QA tests, which give us the possibility of substituting RCF with a diode array. QA would be performed faster than using the film procedure, obtaining reliable results. Regarding the MLC QA, the inability to detect systematic displacements make it difficult to confidently use the detector.

## 1 INTRODUCTION

The CyberKnife system (Accuray, Sunnyvale,  California, USA) is a platform designed for stereotactic radiosurgery (SRS) and stereotactic body radiation therapy (SBRT) that is able to deliver multiple non‐coplanar beams thanks to a robotic arm to which a linac is mounted. An imaging system consisting of two orthogonal x‐ray systems installed in the treatment room allows for intrafraction monitoring and tracking.^1^ Up to now, three different collimator systems are available: fixed collimators, Iris variable collimator, and the Incise MLC.^2^ Fixed collimators consist of 12 circular collimators with diameters from 5 to 60 mm projected at 800 mm source‐axis distance (SAD). The Iris collimator consists of two hexagonal diaphragms that shape almost circular beams with the same nominal field sizes as those available for fixed collimators.^3^ The MLC has 26 leaf pairs, each with a width of 3.85 mm at 800 mm SAD. The maximum field size is 120 × 100.1 mm^2^, while the minimum field size is 7.6 × 7.7 mm^2^.

For treatments to be accurately delivered, the coordinate system of the robotic arm should be perfectly matched to that of the imaging system. Besides, those dynamic collimation systems (cone, Iris and MLC) should be checked periodically in order to ensure that there is no change in the beam geometry. Therefore, a wide QA program must be developed to ensure the correct delivery of treatments. For this purpose, some QA protocols have been developed by Medical Physics Societies.^4^, ^5^ Besides, many of the QA procedures are established by the vendor in which radiochromic films (RCFs) are essential. Thus, it would be desirable to develop some alternative methods that would avoid the use of films, which is time and resource consuming. Recently, high‐resolution arrays have been developed for patient‐specific quality assurance (PSQA) of SRS and SBRT treatments and have proved their usefulness for this purpose.^6^, ^7^, ^8^, ^9^, ^10^, ^11^ Geometric and accuracy tolerances of the CyberKnife system are usually within the submillimeter range. Thus, whether a detector array has the sensitivity and resolution to discriminate such small errors should be investigated.

In this work, we will study the performance of the SRS Mapcheck (Sun Nuclear, Florida, USA) diode array for QA purposes on the CyberKnife system thanks to a new CyberKnife QA module that has been recently developed by Sun Nuclear in the SNC Patient software (version 8.5.1). Tests developed are comprised of a test of daily geometrical accuracy of the CyberKnife system (AQA), a test for field sizes of the variable aperture iris collimator, and a Garden Fence test of the MLC system.^12^ In this work, we evaluate and compare the performance of the SRS Mapcheck array with the methods originally proposed by the vendor based on film dosimetry.

## 2 MATERIALS AND METHODS

### 2.1 SRS Mapcheck detector and SNC Patient software

The SRS Mapcheck (Sun Nuclear, Florida, USA) consists of 1013 *n*‐type diodes with a measurement area of 0.48 × 0.48 mm^2^. The center to center distance is 2.47 mm in the diagonal direction. To allow for localization with the CyberKnife, the detector array is equipped with four built‐in fiducials. Measurements performed with the detector can be analyzed with the SNC Patient 8.5.1 software. The software was originally designed for PSQA, but a new CyberKnife QA tool allows for several QA tests. Details of these QA measurements analysis by the software are not known. Those tests will be described in the following sections.

### 2.2 AQA (AUTO QA)

AQA is a daily test analogous to the Winston‐Lutz test^13^ performed in a standard C‐arm linac. The aim of this test is to check the targeting accuracy of the treatment by pointing an anterior‐posterior (AP) and a lateral (LAT) beam to the given target. The tolerance of this test is a radial error < 0.95 mm.

#### 2.2.1 Vendor procedure

In the vendor procedure, a cubic phantom with four fiducial markers and an acrylic ball in its center is employed. The phantom allows for the placement of two RCF in the coronal and sagittal planes. After performing a computed tomography (CT) of the phantom, a plan is created in which the four fiducial markers are used for localization purposes and the acrylic ball is used as a target. Only an AP and a LAT beam are created, both of them aiming to the ball center. For delivery, an inner acrylic ball is substituted by a metal ball. Thus, the metal ball creates a shadow for each beam on each of the films, permitting the target accuracy of the system to be assessed by analyzing the ball location within each beam. The analysis is performed by a vendor‐provided software.

#### 2.2.2 Array procedure

**FIGURE 1:**
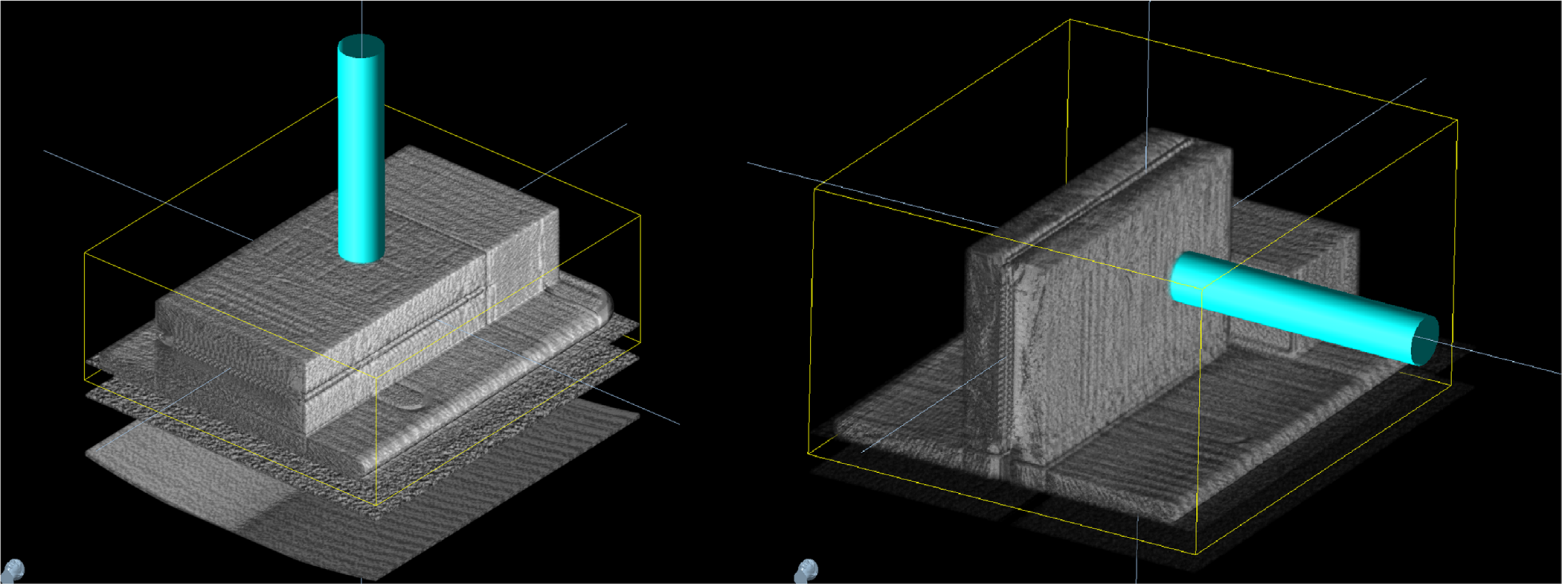
Set up for array AQA irradiation. These two images belong to the different plans needed for this test.

The procedure with the array consists of the creation of two plans that deliver 100 MU to its central detector. In one plan, the array is positioned horizontally with respect to the couch while an AP beam is used. In the second, the array is turned 90 degrees, so a LAT beam intersects perpendicular to the array surface. Both set up are shown in Figure 1. Sun Nuclear provides two virtual CT with associated set of structures, so central detector can be located in the real CT by performing an accurate rigid fusion.

To evaluate the performance of the array, two different tests were performed. Firstly, daily tests were performed with both methods and their results were compared. Secondly, known errors were introduced in the robot AQA path calibration to check for the sensitivity of both methods to those changes. Errors of 0.25, 0.50, and 0.75 mm were introduced in the superior‐inferior (SI) direction (common to both beams), the LAT direction (only detectable in the AP beam), and for the AP and LAT directions simultaneously. Linear fits of both methods were obtained for each direction and for the Radial error, this allows for the measurement of how far are both methods from the ideal detector, which should yield linear relationships with slopes equal to 1.

### 2.3 Iris quality assurance (IRIS QA)

Iris QA is a test designed to check the constancy of Iris collimator field sizes. Baseline values of field sizes must be taken in order to be compared with measurements in the future. According to the vendor, the field sizes should be within a 0.2 mm change from the baseline value. Also, the QA protocol proposed by the Canadian Organization of Medical Physicists (COMP)^5^ establishes a tolerance level of ± 0.3 mm and an action level of ± 0.5 mm. This test should be performed on a monthly basis.

#### 2.3.1 Vendor procedure

A RCF is placed into a plastic mount consisting of a base plate and a 15 mm build‐up plate. Then, the mount is inserted on an accessory (birdcage) attached to the robot head, which ensures that the film is at 800 mm SAD. 600 MU are delivered to the film using the field size of interest. Thereafter, the film must be scanned and analyzed with a vendor‐provided software. In order to perform the measurement, the software needs a blank film to correct for the background and four films irradiated with the 15 mm fixed cone to obtain the pixel value defining the field width. This should be made before establishing a baseline for each field size.

#### 2.3.2 Array procedure

While the vendor procedure requires that all measurements be performed in the Physics Mode of the CyberKnife system, the array procedure requires a clinical plan. This way, a CT scan of the detector is employed to create a plan in which 11 beams with field sizes from 7.5 to 60 mm deliver 100 MU. All beams are delivered and measured at the same time. The CyberKnife QA tool is then able to extract temporal data from the measurement file and analyze each field separately. The QA of the 5 mm field is not supported, as it involves too few detectors in the high dose region of the beam.

To evaluate the performance and sensibility of the SRS Mapcheck to field size changes, the service mode of the CyberKnife system was employed to include known errors to the clinical fields by manually selecting the field size value. Changes in the clinical aperture from −0.5 to 0.5 mm in steps of 0.1 mm were included. Considering that each measurement was repeated four times and that 11 field sizes can be evaluated, 484 total measurements were performed and analyzed.

### 2.4 MLC QA

This QA task is intended to check the MLC positioning accuracy. It is performed with a Garden Fence test that consists of the irradiation of five strips with gaps between each pair of strips. The mean position of each bank is compared with the expected position. In addition, the deviation of single leaves is also checked. This test is part of the monthly QA of the CyberKnife system. Test criteria defined by the vendor are as follows:

1. Mean bank offset error should be within 0.2 mm.
2. All leaf positions must be within 0.95 mm of the expected value.
3. Each bank must have no more than 13 leaf junctions with a deviation higher than 0.5 mm.
4. No more than 1 position per leaf should have a deviation larger than 0.5 mm from the expected position.

#### 2.4.1 Vendor procedure

The vendor procedure consists of placing a RCF in a holder (MLC QA tool) that is attached to the robot head. This holder is equipped with two radio‐opaque markers that are used as a reference for alignment and centering of the beam in the film. Then, a Picket Fence pattern in which five 10 mm strips with 15 mm gaps are irradiated with 170 MU/strip. The film is scanned with a resolution of 600 dpi. Before the analysis, a rotation correction is performed to the image based on the shadow produced by the markers of the holder. The analysis is performed using the MLC QA module of RIT 6.7 software (Radiological Imaging Technology, Colorado Springs, Colorado, USA). This software needs a template of the irradiated pattern to perform the analysis. It should be noted that while the film is placed at 433.5 mm from the focus, all tolerances and technical specifications are provided at 800 mm.

#### 2.4.2 Array procedure

To perform this procedure, the array must be scanned with its long axis rotated 90 degrees with respect to the longitudinal axis of the treatment couch. Then, three clinical plans are created in the treatment planning system (TPS).

1. The first plan is exclusively used for correctly aligning the array detector. In this plan, the align center is placed in the center of mass of the four build‐up fiducial markers of the array, making it possible to correct for rotations.
2. The two other plans are used for delivery of the Garden Fence pattern. Due to the limited size of the array (77 x 77 mm^2^), it is not possible to analyze all 26 leaf pairs in one plan.
3. The second plan performs the garden fence on leaves 1 to 13, while the third does it on leaves 14 to 26. For each plan, the array detector is displaced accordingly to center the delivery pattern on it. Also, the align center of these plans is moved a distance of 72.73 mm toward the linac.

**FIGURE 2:**
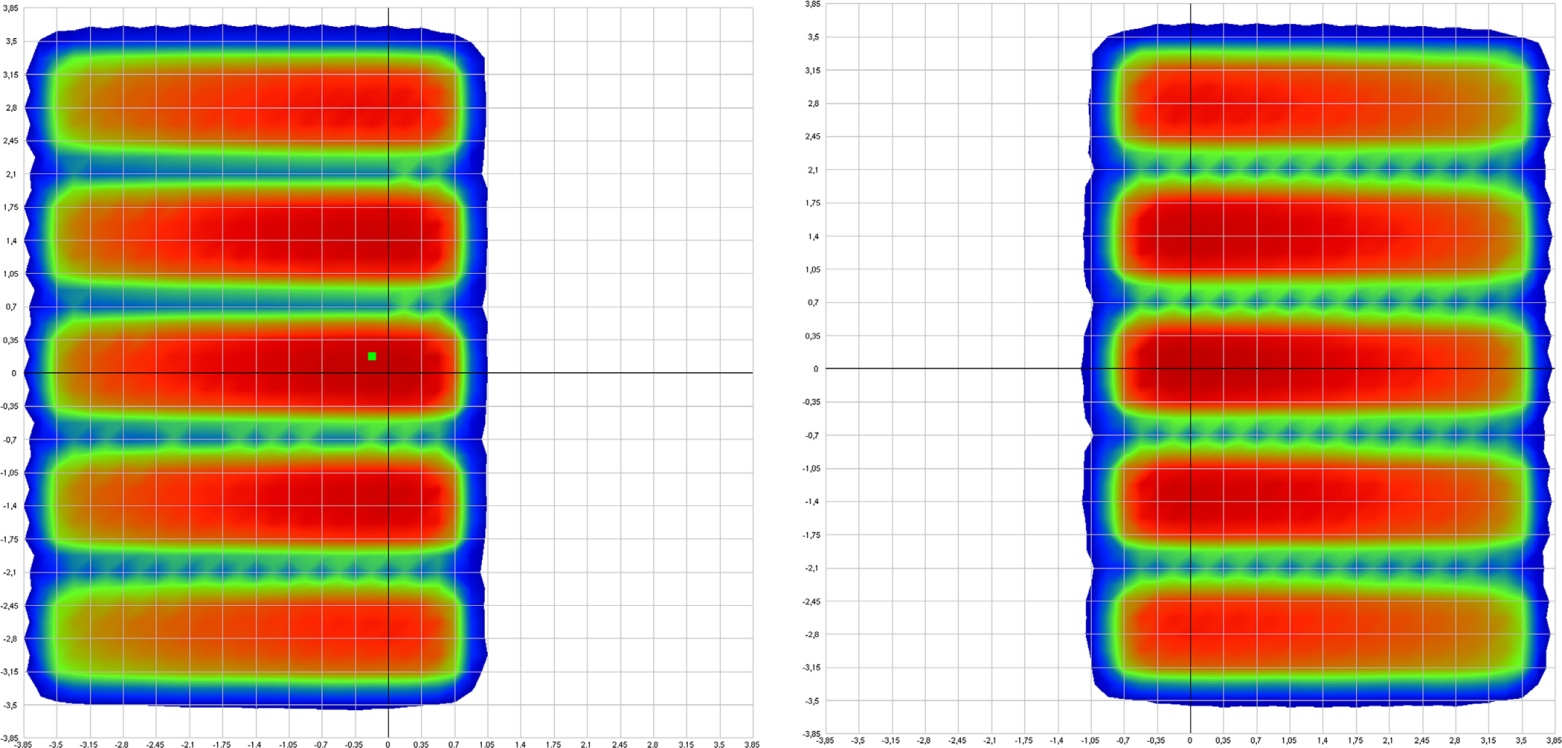
Garden fence pattern irradiated in the array. Each image refers to a different irradiation plan. The left one is defined by leaves 1 to 13, while the right does it using leaves 14 to 26.

The Garden Fence pattern will consist of five strips with variable widths of 11.5−11.6 mm and a gap between the strips of 3.8−3.9 mm as required by vendor user's guide. Strip patterns irradiated in the array detector are shown in Figure 2.

Both measured patterns are analyzed automatically by the CyberKnife Machine QA tool according to the criteria indicated above.

It should be noted that the procedure proposed by Sun Nuclear is dependent on the definition of the align center in the TPS. Thus, it is not able to detect systematic errors in the leaf positioning. This fact is advised in the User Guide of the software.

To evaluate the performance of the array, two different tests were performed. Firstly, results obtained with the array were compared with the standard procedure based on RCF. Secondly, modifications in the clinical plans used to perform the MLC QA were introduced.

To check the potential of the array to detect systematic errors in the leaf bank positioning, four different plans were created with systematic deviations in both the X1 and X2 leaf banks. Displacements ranged from −0.4 to +0.4 mm in steps of 0.1 mm. Each bank was shifted a different amount at each plan, with a difference of 0.1 mm between them. That is, in Plan 1, the X1 bank was shifted −0.1 mm and the X2 bank was shifted −0.2 mm. In Plan 2, the shift applied to X1 was −0.3 mm and to X2 was −0.4 mm and so on, until all shifts from −0.4 to +0.4 mm were covered.

Lastly, to analyze the performance of the array in detecting individual leaf errors, two plans with errors of ± 0.8 and ± 1.2 mm in certain leaves were created and delivered.

## 3 RESULTS

### 3.1 AQA

#### 3.1.1 Daily tests

Fourteen daily tests with the fixed collimator and 17 with the MLC collimator were compared. As baseline values from each methods were taken on different dates, data from the first day of measurements were taken as a baseline. The mean value and standard deviation of the differences of both methods for both collimators are shown in Table 1. Systematic deviations of up to 0.20 mm are obtained between both methods, and standard deviations are below 0.15 mm. If the analysis is performed for each detector separately, higher repeatability is obtained with the SRS Mapcheck detector than with the films. The SI direction shows a better repeatability for both methods, as this direction can be measured in the two projections employed.

**TABLE 1:** Mean value and standard deviation of the results obtained from the daily tests performed with the SRS Mapcheck and with the film. Mean value of the daily difference between both methods is also shown.

	Fixed (*n* = 14)			MLC (*n* = 17)		
Mean values (mm) (SD)	LAT	AP	SI	LAT	AP	SI
**SRS Mapcheck (SD)**	−0.02 (0.08)	0.06 (0.07)	−0.02 (0.04)	0.03 (0.08)	−0.05 (0.08)	−0.05 (0.04)
**Film (SD)**	−0.14 (0.13)	−0.04 (0.11)	0.11 (0.10)	−0.15 (0.14)	0.01 (0.09)	0.01 (0.06)
**Difference (SD)**	−0.12 (0.14)	−0.1 (0.12)	0.14 (0.11)	−0.18 (0.14)	0.06 (0.13)	0.06 (0.06)

Abbreviations: AP, anterior‐posterior; LAT, lateral; SD, standard deviation; SRS, stereotactic radiosurgery.

#### 3.1.2 Known errors

**FIGURE 3:**
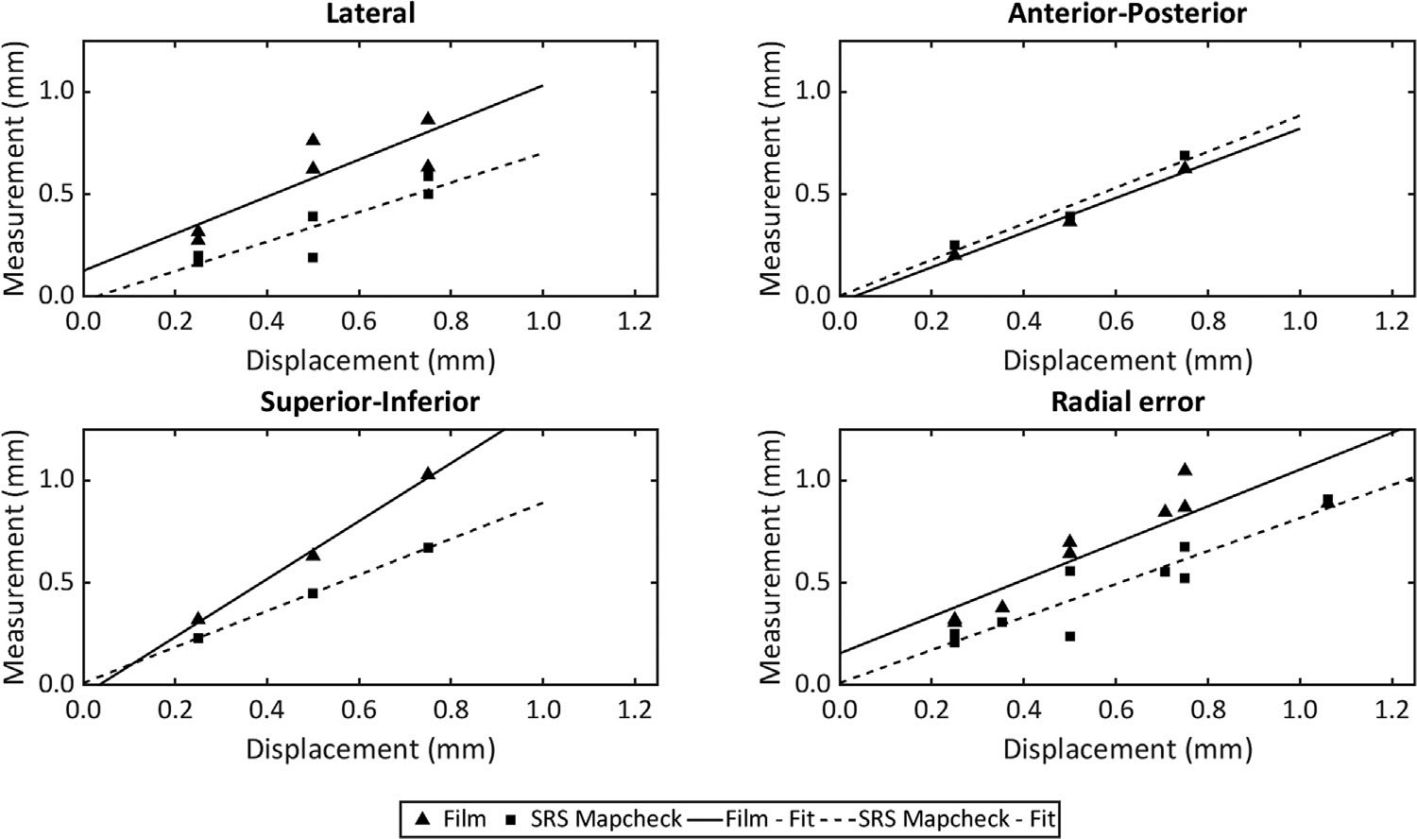
Relationship between the set error and measured displacement for the AQA test at each direction and for the radial error with both methods. Linear fits are also shown.

By changing the Robot calibration for the AQA plans, it was possible to assess the performance of both methods in detecting calibration errors. The results are shown in Figure 3, where the relationship between the applied displacements and measurements are shown for each direction. SRS Mapcheck procedure seems to report smaller shifts than expected in all cases. Radial errors for all the measurements performed are also shown. Parameters of the linear fits shown in Figure 3 are represented in Table 2. The worst correlations were found for both methods in the lateral directions, which affect the correlations measured for the Radial error. In the other directions, the values of *r* ^2^ are very close to 1 in both cases. The intercept is very close to 0 in the directions with better goodness of fit (SI and AP), while for the Lateral and Radial error, the array shows lower values. The slopes are similar for both methods in most of the cases, except for the SI direction, where the film has a slope of 1.4 while the array has a value of 0.88.

**TABLE 2:** Parameters of the linear fits shown in Figure 3. *a* represents the slope and *b* represents the intercept of the relationship between the set and measured errors.

Linear fit equation: Measurederror=a.Seterror+b												
	Lateral			Anterior‐posterior			Superior‐inferior			Radial error		
	*a*	*b*	*r* ^2^	*a*	*b*	*r* ^2^	*a*	*b*	*r* ^2^	*a*	*b*	*r* ^2^
Film	0.9	0.12	0.73	0.85	−0.03	0.98	1.4	−0.05	0.995	0.9	0.15	0.79
SRS Mapcheck	0.72	−0.02	0.8	0.88	0	0.96	0.88	0.01	1	0.8	0.01	0.85

Abbreviations: SRS, stereotactic radiosurgery.

#### 3.1.3 Iris QA

**FIGURE 4:**
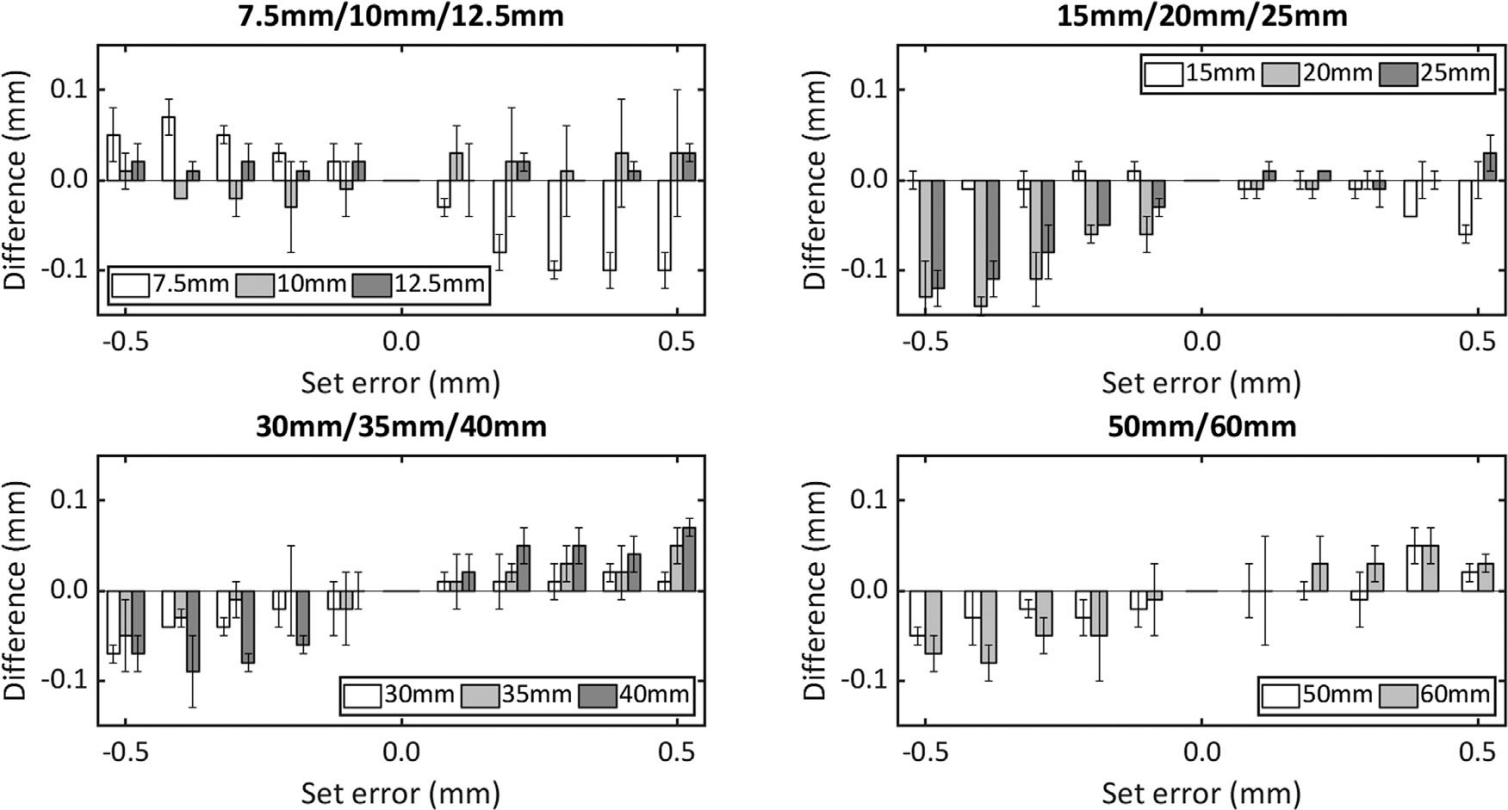
Mean residual error of the measurements when certain errors are set for the 11 field sizes available for analysis with SRS MapCheck error bars representing 1σ of each measurement. The set error range is from −0.5 to +0.5 mm in steps of 0.1 mm. SRS, stereotactic radiosurgery.

Firstly, measurements were grouped according to the radiation field clinical size. The comparison between the expected and measured differences is shown in Figure 4.

As measurements of modified fields were compared with those corresponding to the clinical field size, a linear regression through the origin could be calculated. The results of these regressions are shown in Table 3.

**TABLE 3:** Linear regression slope and *r* ^2^ for each field size.

Field size (mm)	7.5	10	12.5	15	20	25	30	35	40	50	60
Slope	1	1.05	1	0.96	1.15	1.14	1.08	1.08	1.17	1.07	1.13
*r* ^2^	1.000	0.998	0.999	0.996	0.979	0.989	0.997	0.999	0.996	0.998	0.998

Linear regressions show that the measured data behave highly linearly. A slope of 1 would represent an ideal response to known field size changes. Although slopes are slightly higher than 1, that disagreement with the ideal response can be useful in order to detect changes in the Iris field sizes.

Measurements can instead be grouped by nominal difference without paying attention to the original clinical size. Forty‐four measurements were taken for each modification. The summary is tabulated in Table 4.

**TABLE 4:** Nominal and measured differences when known errors are set.

Nominal difference (mm)	−0.5	−0.4	−0.3	−0.2	−0.1	+0.1	+0.2	+0.3	+0.4	+0.5
Measured difference (mm)	−0.55	−0.45	−0.33	−0.22	−0.11	0.10	0.21	0.30	0.41	0.51
SD (mm)	0.06	0.06	0.05	0.04	0.03	0.03	0.04	0.04	0.04	0.05

Abbreviations: SD, standard deviation.

To verify the statistical significance of these differences, each set of differences was compared with their immediately smaller and larger set. A *t*‐test was performed with a significance level of 5% for each pair of sets. All comparisons were found to be significant (*p* < 0.05).

#### 3.1.4 MLC QA

As stated above, five MLC QA tests were performed with both methods (SRS Mapcheck and RCF). The summary of the results is shown in Table 5.

**TABLE 5:** Summary of the five tests measured per method. Mean Bank Offset (MBO), number of leaves with deviations higher than 0.95 and 0.5 mm, and whether the test met the passing criteria are shown.

	Radiochromic film					Diode array				
N	MBO X1 (σ) (mm)	MBO X2 (σ) (mm)	*N*° > 0.95	*N*° > 0.5 (repeat)	Pass test	MBO X1 (σ) (mm)	MBO X2 (σ) (mm)	*N*° > 0.95	*N*° > 0.5 (repeat)	Pass test
1	−0.02 (0.06)	−0.02 (0.06)	0	0 (*n*)	y	0.03 (0.15)	0.02 (0.14)	0	1 (*n*)	Y
2	−0.03 (0.10)	0.0 (0.4)	1	0 (*n*)	N	0.01 (0.11)	0.00 (0.12)	0	0 (*n*)	Y
3	0.00 (0.10)	0.02 (0.13)	1	0 (*n*)	N	−0.03 (0.07)	−0.03 (0.10)	0	0 (*n*)	Y
4	−0.01 (0.09)	0.1 (0.7)	2	1 (*n*)	N	0.01 (0.13)	0.00 (0.12)	0	0 (*n*)	Y
5	0.03 (0.07)	0.06 (0.06)	0	0 (*n*)	y	0.00 (0.09)	−0.01 (0.11)	0	0 (*n*)	Y

The results of the Mean Bank Offset for both methods are close to 0, and their standard deviations are also very similar (around 0.1 mm). Using the diode array, all five tests passed all criteria. Meanwhile, with the RCF, three of them failed due to deviations higher than 0.95 mm in at least one leaf position. The failing tests showed errors of 4.3, 4.6, 5.7, and 1.1 mm in only one position of different leaves. The largest errors would be visible to the naked eye, so the main reason for these measurements are spots of dust that distort the analysis. In the case of the 1.1 mm error, it was not found in further measurements, so the main hypothesis is that it was caused by a flaw in the film rather than in the MLC.

The vendor procedure does not allow known variations to be introduced either to the whole bank or to a single leaf. The procedure is performed in physics mode and this option is not available.

When systematic deviations were added to whole MLC banks, the SNC Patient software was not able to detect them, as it is designed to ignore them in order to handle alignment errors from the align center in the delivery plan.

Nevertheless, raw data from those plans with errors were compared with another delivery without intended errors used as a reference. That way, it was possible to analyze the ability of the detector to catch systematic errors banks of leaves.

The results obtained when measuring the plans with known errors and comparing them to a reference are shown in Table 6. Each plan applied a different error to X1 and X2 banks until the range from −0.4 to 0.4 mm was fulfilled.

**TABLE 6:** Results for mean bank offsets detected with the diode array without applying the alignment correction when compared with a reference plan.

	Plan 0 (reference)		Plan 1		Plan 2		Plan 3		Plan 4	
	X1	X2	X1	X2	X1	X2	X1	X2	X1	X2
Planned (mm)	0.00	0.00	+0.10	+0.20	+0.30	+0.40	−0.10	−0.20	−0.30	−0.40
Measured (σ) (mm)	0.00	0.00	0.23	0.22	0.54	0.53	−0,23	−0.22	−0.50	−0.47
	(ref)	(ref)	(0.07)	(0.01)	(0.01)	(0.01)	(0.11)	(0.10)	(0.01)	(0.01)

It is noted that when tracing results to a reference, this procedure is able to detect shifts in MLC banks. Although numerically it does not exactly match what is expected, this would help to detect systematic positioning errors.

When applying positional errors to individual leaves, the detector was also able to detect them, as shown in Table 7, where the measured values of shifts are shown and compared to the true values. As the errors were applied to more than one leaf position, the mean values and their standard deviations were calculated. The mean difference between both values was, in all cases, less than 0.4 mm, and a potential of the array to detect errors of 0.5 mm seems feasible in most cases. Also, their standard deviations tend to be small, suggesting a uniformity of the array detectability in all the range of leaf positions.

**TABLE 7:** Leaf numbers 1, 2, 8, 9, 23, and 24 have known deviations in all strips, while leaves 12, 13, 14, 15, 18, and 19 have known deviations in two out of the five strips. Results were measured with SRS Mapcheck.

Leaf n°	1	2	8	9	12	13	14	15	18	19	23	24
Planned (mm)	+0.8	+0.8	+0.8	+0.8	+1.2	+1.2	−1.2	−1.2	+1.2	+1.2	−0.8	−0.8
Measured (σ) (mm)	1.1	0.6	0.4	0.7	0.6	1.0	−1.07	−1.10	1.0	1.2	−0.7	−0.8
	(0.1)	(0.1)	(0.1)	(0.1)	(0.2)	(0.3)	(0.03)	(0.02)	(0.2)	(0.1)	(0.1)	(0.1)

## 4 DISCUSSION

In this work, we have tested a new tool for CyberKnife QA based on measurements with the SRS MapCheck array.

The main advantage of the use of a diode array for QA of the CyberKnife system is to avoid the uncertainties involving RCF dosimetry. Besides, the results obtained with the diode are available almost immediately once the measurement is performed. On the other hand, the spatial resolution of the array is far lower than that of RCF, which, given the tight tolerances involving CyberKnife geometrical tests, impose an in‐depth investigation of the capabilities of the array. Thus, the feasibility of those and vendor procedures are based on them. In this context, the results for all available tests using the CyberKnife QA tool included in the SNC Patient software will be summarized in the following sections and compared with vendor procedures.

In the case of the AQA test, it is shown that differences between daily tests with both methods yield differences below 0.2 mm. Taking into account that reproducibility was better with the array detector and, for known errors, the behavior of both methods is similar, showing a slightly higher linearity in the case of the array, application of the method with the array in daily practice would be feasible, as it avoids the use of a large amount of RCF and less manipulation is needed to obtain results. Similar results have been found in both procedures. The procedure with film has lower reproducibility, which can be attributed to the film handling and scanning procedure. Automatic alignment in the array procedure makes it almost operator‐independent. Therefore, we think the array procedure is a reliable substitute for the RCF procedure.

For the Iris QA, it is shown that the array faithfully reproduces changes in the size of the Iris collimation system, managing to detect variations of ± 0.1 mm. Considering the tolerances involved in this test (0.2−0.3 mm), it can be stated that the new method developed by Sun Nuclear is appropriate for monthly verification of Iris apertures. The new system makes it possible to check all available openings with a single measurement, which indeed reduces the time requirements compared to the vendor procedure, both in the treatment unit and in further processing.

Regarding the QA of the MLC, the array procedure gives comparable results to those of RCF and shows the capability of detecting single leaf deviations. However, software alignment correction performed by the software to handle alignment errors coming from the TPS could mask systematic shifts of a whole bank. Therefore, the vendor procedure with RCF should still be considered the standard procedure for QA of the MLC. It has been shown that this pitfall could be partially overcome if the software was to allow a reference to trace the measurements to, as it could then be capable of detecting systematic bank shifts. Nevertheless, the procedure proposed by Sun Nuclear is cumbersome, implying a longer delivery time than the standard procedure with film. Furthermore, Sun Nuclear proposes this procedure as a daily test, hindering the application of this procedure, as the actual daily test is based on a visual inspection of a Picket Fence pattern delivering a homogeneous dose.

## 5 CONCLUSIONS

The SRS Mapcheck detector was sensitive and accurate for detecting geometrical changes in both AQA and Iris QA tests and was able to do it faster and while saving resources such as RCF. Regarding the MLC QA test, the inability of the actual software to detect systematic errors in leaf positions, added to the fact that the proposed procedure is more or equally time consuming than the actual procedure based on film, makes it difficult to apply the use of the detector for the QA of CyberKnife MLC in the clinical practice.

## AUTHOR CONTRIBUTIONS

Juan D. García‐Fuentes and David Sevillano have developed the idea, designed and performed the measurements, analyzed the results and written this manuscript. Rafael Colmenares, Ana B. Capuz, Rafael Morís, Miguel Cámara, Pablo Galiano, Sandra Williamson, María J. Béjar and Daniel Prieto contributed gathering data and analysis. Feliciano García‐Vicente performed critical revision of the manuscript.

## CONFLICT OF INTEREST STATEMENT

The authors declare no conflicts of interest.
